# 

**Published:** 1845-07

**Authors:** 


### CONTENTS

OF NO. I.

### ORIGINAL COMMUNICATIONS.

### ESSAYS AND CASES.

Art. I.—Some account of Erysipelatous Fever as it occurred in Whitesburg and its vicinity, Madison county, Ala., during the Spring and Summer of 1844. By Preston Capshaw, M.D., of Whitesburg, Ala. - - - - 1

Art. II.—A Case of Imperforate Vagina. Reported by W. L. Sutton, M.D., of Georgetown, Ky. - - - 11

Art. III.—A Case of Injury of the Hip by a fall, with exfoliation of the bone. By John M. Ross, M.D., of Marion, Mississippi. - - - - - - - - - 14

Art. IV.—Lectures on Diseases of the Chest. By J. R. Buck, M.D., Lecturer on the Theory and Practice of Medicine in the Louisville Summer School of Medicine. - - - - - - - - - - - 16

Art. V.—A Case of Phrenitis resulting in Death, from the Inhalation of Sulphuric Ether. By Dr. J. Miller, of Louisville, Ky. - - - - - - - - 25

### REVIEWS.

Art. VI.—Twelfth Annual Report of the Trustees of the State Lunatic Hospital at Worcester. - - - - - 31

Art. VII.—A Treatise on the Diseases and Special Hygiene of Females. By Colombat De L'Isere. Translated from the French, with additions. By Charles D. Meigs, M.D., Professor of Midwifery and the Diseases of Women and Children in Jefferson Medical College, Philadelphia; Member of the American Philosophical Society; of the Philadelphia College of Physicians; of the Philadelphia Medical Society; and Physician to the Lying-in Department of the Pennsylvania Hospital. - 37

SELECTIONS FROM AMERICAN AND FOREIGN JOURNALS

A Lecture on the Modern Improvements of Surgery. - - 61
Unusual form of Intus-Susceptio of the Colon. - - - 80
Fumes of Nitrate of Potass in Spasmodic Asthma. - - 84

ORIGINAL INTELLIGENCE.

Quackery. - - - - - - - - - - - - 85
Typhoid Fever in Missouri. - - - - - - - - 88
Louisville Summer School of Medicine. - - - - - 89
Death of Professor Sewall. - - - - - - - - 89
Prize Essay. - - - - - - - - - - - 90
Medical Society of Tennessee. - - - - - - - 90
Professor Dickson on the Intermarriage of the races. - - 91
Bloodless Amputations. - - - - - - - - - 92

